# A review of the Nearctic genus *Zealeuctra* Ricker (Plecoptera, Leuctridae), with the description of a new species from the Cumberland Plateau region of eastern North America

**DOI:** 10.3897/zookeys.344.5912

**Published:** 2013-10-22

**Authors:** Scott A. Grubbs, Boris C. Kondratieff, Bill P. Stark, R. Edward DeWalt

**Affiliations:** 1 Department of Biology and Center for Biodiversity Studies, Western Kentucky University, Bowling Green, Kentucky, 42101, USA; 2 Department of Bioagricultural Sciences and Pest Management, Colorado State University, Fort Collins, Colorado, 80523, USA; 3 Department of Biology, Mississippi College, Clinton, Mississippi, 39058, USA; 4 University of Illinois, Prairie Research Institute, Illinois Natural History Survey, 1816 S Oak St., Champaign, Illinois, 61820, USA

**Keywords:** Plecoptera, Leuctridae, *Zealeuctra*, new species, North America

## Abstract

The stonefly genus *Zealeuctra* (Plecoptera: Leuctridae) is endemic to the central and eastern Nearctic regions and is presently comprised of 10 species. Scanning electron microscopy (SEM) was used to examine and redescribe two important diagnostic features typically used to identify and define the adult male stage: the large, anteriorly-recurved epiproct and the medial cleft of the ninth abdominal tergite. SEM was also employed to depict the posteromedial portion of female 7^th^ sternum. A new species, *Z. ukayodi* **sp. n.**, is described from the Cumberland Plateau region of northeastern Alabama and Tennessee. The new species appears superficially similar to *Z. talladega* Grubbs, but is easily differentiated by characteristics of the male medial cleft. An updated taxonomic key to the males of *Zealeuctra* is provided.

## Introduction

The subgenus *Zealeuctra* Ricker, 1952 was erected to include *Leuctra claasseni* Frison (Ricker 1952). Illies (1966) later elevated *Zealeuctra* to full generic rank. *Zealeuctra* remained monotypic until the comprehensive study of Ricker and Ross (1969). Six new species were described: *Zealeuctra arnoldi* Ricker & Ross, 1969, *Zealeuctra fraxina* Ricker & Ross, 1969, *Zealeuctra hitei* Ricker & Ross, 1969, *Zealeuctra narfi* Ricker & Ross, 1969, *Zealeuctra wachita* Ricker & Ross, 1969, and *Zealeuctra warreni* Ricker & Ross, 1969. Poulton and Stewart (1991) subsequently described the male of *Zealeuctra wachita* since the original description of this species was based only on the female. Three additional species have since been described: *Zealeuctra cherokee* Stark & Stewart, 1973, *Zealeuctra stewarti* Kondratieff & Zuellig, 2004, and *Zealeuctra talladega* Grubbs, 2005 (Stark and Stewart 1973; Kondratieff and Zuellig 2004; Grubbs 2005).

*Zealeuctra* is endemic to the central and eastern Nearctic regions, and is typically associated with intermittent or temporary upland streams (Snellen and Stewart 1979; Stewart and Stark 2002). *Zealeuctra claasseni* and *Zealeuctra fraxina* are the only species distributed broadly. In contrast, three species are found mainly in the Texas Hill Country region (*Zealeuctra arnoldi*, *Zealeuctra hitei*, and *Zealeuctra stewarti*), three species are regional endemics within the Interior Plateau region (*Zealeuctra cherokee*, *Zealeuctra wachita*, and *Zealeuctra warreni*), *Zealeuctra narfi* is distributed from Arkansas and Missouri north to Wisconsin, and *Zealeuctra talladega* is known only from the Talladega Mountains region in eastern Alabama.

*Zealeuctra* exhibits several unique characteristics in the adult stage, namely the swollen male cerci with accessory humps and/or spines, the male ninth tergite bearing a conspicuous medial depression (i.e. “cleft”), and the female seventh sternite having a posteromedial lobe (although secondarily lost in two species; Ricker and Ross 1969). The two diagnostic features typically used to identify and distinguish between males are the (1) large, anteriorly-recurved epiproct and the (2) shape and sclerotization patterns of the cleft. The shape and arrangement of cercal lobes/spines can also aid with identifications (e.g. Poulton and Stewart 1991). In addition, the fused subanal plates-anal probe structure appears to offer diagnostic information yet this has not been fully studied. Identifying females to species is markedly easier if associated males are present. Females are identified by the hind margin of the seventh abdominal sternite, notably the (a) presence or absence of a central notch and lobe and (b) shape of the “shoulders” if a notch is present. *Zealeuctra* nymphs are uncommon in collections and only one species has been described in detail (*Zealeuctra claasseni*; Stewart and Stark 2002).

In this study scanning electron microscopy (SEM) was employed to examine two diagnostic features of *Zealeuctra* males, focusing on the epiproct and the abdominal cleft. The posteromedial portion of the female seventh abdominal segment is also depicted with SEM micrographs. A new species is described herein and an updated taxonomic key to the males of *Zealeuctra* is provided.

## Materials and methods

Most of the *Zealeuctra* specimens examined in this study were obtained from the Monte L. Bean Museum, Brigham Young University, Provo, Utah, USA (BYUC), C.P. Gillette Museum, Colorado State University, Fort Collins, Colorado, USA (CSUC), S.A. Grubbs collection, Western Kentucky University, Bowling Green, Kentucky, USA (WKUC), B.P. Stark Collection, Mississippi College, Clinton, Mississippi, USA (BPSC), and the Illinois Natural History Survey, Champaign-Urbana, Illinois, USA (INHS). Other codens used were TAMU (Texas A&M University Insect Collection, College Station, Texas, USA), and USNM (National Museum of Natural History, Smithsonian Institute, Washington D.C., USA). Location data (in decimal degrees) for each specimen record were recorded either directly with portable GPS units or georeferenced from vial label data (if possible).

Specimens for SEM analyses were dehydrated through a series of 75%, 90%, 95%, and 100% ethanol for 10 minutes each, and placed in Hexamethyldisilizane for 30 minutes. Dehydrated specimens were attached to aluminum stubs with double-stick tape and coated with gold-palladium using an Emscope SC500. Coated specimens were examined using a Jeol JSM-5400LV scanning electron microscope and digital images were captured with an IXRF system.

## Results and discussion

Ricker and Ross (1969, their figure 29) placed *Zealeuctra* in a polytomy with *Paraleuctra* Hanson, 1941, *Rhopalopsole* Klapálek, 1912, and *Leuctra divisa* Hitchcock, 1958 (the latter is now included in *Paraleuctra* (Stark & Kyzar 2001)). They postulated that these taxa were grouped by two synapomorphies: females with an incomplete 10^th^ abdominal sternite and the presence of membranous pleural folds on larval abdominal segments 1–6 (shared also by *Moselia* Ricker, 1943, (Stewart and Stark 2002)). Ricker and Ross (1969, their figure 28) proposed that the seven species of *Zealeuctra* recognized at that time were derived from a series of three basal ancestors, and that *Zealeuctra narfi* was the most ancestral species.

Testing Ricker and Ross’s (1969) hypotheses, however, and assessing how those species described since 1970 fit within an evolutionary framework is mostly beyond the scope of this paper. This would require a comparative morphological assessment of the fused subanal plates-anal probe structure, and arguably more importantly, a modern and robust phylogenetic analysis using molecular techniques (e.g. mitochondrial cytochrome *c* oxidase I gene sequencing).

An updated taxonomic key to the males of *Zealeuctra* is provided, and a new *Zealeuctra* species is described herein from the Cumberland Plateau region of southern Tennessee and northeastern Alabama.

### Key to *Zealeuctra* males, modified in part from Ricker and Ross (1969) and Poulton and Stewart (1991)

**Table:** 

1	Cleft (=medial depression) tapering and V-shaped, especially in anterior half (Figs 3A–D, 5A)	2
–	Cleft distinctly U-shaped in anterior half (Figs 1A, 4A, 9A)	3
2	Epiproct with a broad triangular base (Figs 3E–H); inner margins of cleft bearing several medial crenulations (Figs 3A–D); widespread distribution across the eastern and central USA (Fig. 12)	*Zealeuctra claasseni* (Frison)
–	Epiproct base narrowly triangular and with a minor shelf-like anterior projection (Figure 5B); inner margins of cleft lacking crenulations and with only a single tooth-like medial projection in posterior half (Fig. 5A); known mainly from central Texas (Fig. 11)	*Zealeuctra hitei* Ricker & Ross
3	Distal portion of epiproct with only one spine (Figs 2B, 8D, 9C)	4
–	Distal portion of epiproct comprised of two distinct spines (Fig. 4B) or one prominent spine plus a prominent, accessory posterior cusp (Figs 1C, 10B)	9
4	Epiproct spine very long and slender (Kondratieff and Zuellig 2004, their fig. 2); endemic to south-central Texas (Fig. 13)	*Zealeuctra stewarti* Kondratieff & Zuellig
–	Epiproct spine markedly shorter, either lacking (Figs 2B, 6C, 9C) or bearing a prominent anterior shelf-like projection at base (Fig. 7E–F, 8C–E)	5
5	Epiproct bearing a prominent shelf-like projection at base, either rounded and subquadrate (Figs 6F, 8C–8D) or angular and squarish (Figs 7D–D, 8E)	6
–	Epiproct base not as above (Figs 2B, 6C, 9C)	7
6	Inner margins of cleft sinuous, without crenulations along inner margins (Figs 7A–7C); anterior, recurved portion of epiproct possessing a minute, medially-positioned hump (Figs 7D–F); known only from the southern Talladega Mountains region of eastern Alabama (Fig. 13)	*Zealeuctra talladega* Grubbs
–	Inner margins of cleft V-shaped to almost sinuous, bearing several large crenulations along inner margins on posterior half (Figs 8A–B); anterior, recurved portion of epiproct possessing a medial hump (Figs 8C–E); distributed across the southern Cumberland Plateau region from central Tennessee south to northeastern Alabama (Fig. 13)	*Zealeuctra ukayodi* Grubbs, sp. n.
7	Anterior portion of cleft with a secondary U-shaped extension, with inner margins set apart from remainder of cleft, medial subtruncate thumb-like projections present at terminus of cleft (Fig. 9A); epiproct anteriorly recurved gradually from base to tip (Fig. 9C)	*Zealeuctra wachita* Ricker & Ross
–	Cleft U-shaped, with margins concave and contiguous throughout entire length, two medially-projected terminal processes present at terminus of cleft (Figs 2A, 6A); epiproct recurved abruptly, ca. 90° degree angle anteriorly (Figs 2B, 6C)	8
8	Epiproct with a small, thickened, shelf-like structure posteriorly (Fig. 2B)	*Zealeuctra cherokee* Stark & Stewart
–	Epiproct lacking a posterior thickening (Fig. 6C)	*Zealeuctra narfi* Ricker & Ross
9	Epiproct comprised of two prominent spines, a large posterior spine plus an accessory anterior spine (Fig. 10B)	*Zealeuctra warreni* Ricker & Ross
–	Epiproct bearing only a small, subterminal cusp posterior to the main spine (Figs 1B, 4C)	10
10	Epiproct base bearing a conspicuous, slightly concave cusp anteriorly (Figs 1B–C); known only from Texas (Fig. 12)	*Zealeuctra arnoldi* Ricker & Ross
–	Epiproct base simple and lacking a prominent cusp, at most, only a very small rounded projection anteriorly (Fig. 4C); sporadically distributed across the central and eastern USA (Fig. 11)	*Zealeuctra fraxina* Ricker & Ross

#### Zealeuctra arnoldi

Ricker & Ross

http://species-id.net/wiki/Zealeuctra_arnoldi

Figs 1 , 12

Zealeuctra arnoldi Ricker & Ross, 1969: 1114. Holotype ♂ (INHS), Sorrell Creek, 1 mi S of Hancock, Comal Co., Texas

##### Description.

**Figures 1.**
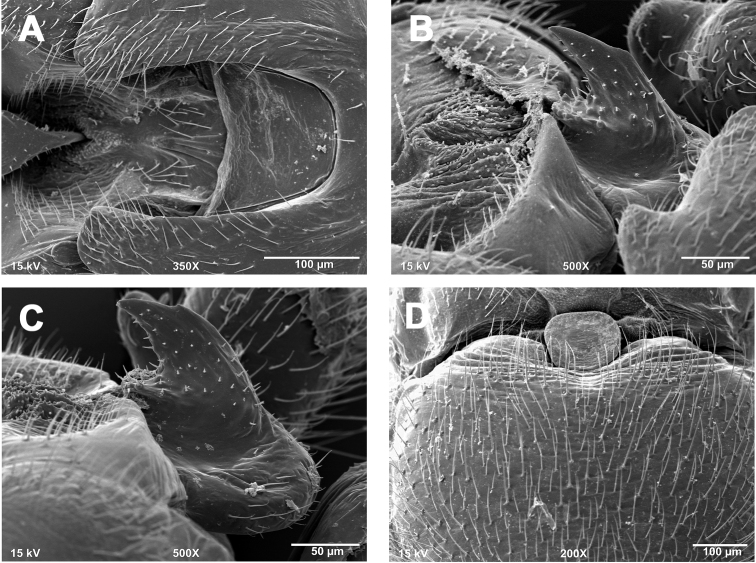
*Zealeuctra arnoldi*, scanning electron micrographs, USA, Texas, Uvalde Co., Cherry Creek, 3 April 2004. **A** male, cleft, dorsal view, 350× **B** male, epiproct, lateral view, 500× **C** male, epiproct, lateral view, 500× **D** female, posteromedial portion of seventh abdominal sternite, 200×.

**Male - epiproct.** Base short and robust, narrowing slightly to a short, recurved, subtriangular spine (Figs 1B–C). A short, slightly concave projection extends anteriorly from the base to approximately the same plane as the spine. Base with bulbous posterior swelling, spine with small accessory posterior cusp that is low and nub-like.

**Female - 7^th^ sternum.** Seventh sternum with a large, subquadrate lobe nested in a distinct, central notch. The notch is bordered laterally by large, convex “shoulders” (Fig. 1D).

**Male - abdominal tergal cleft.** Simple in outline. Anterior portion wide and broadly U-shaped, with a nearly straight terminal margin (Fig. 1A). Posterior portion narrowing slightly with no medial projections and only scarcely visible crenulations along inner margins.

##### Material examined.

**USA**, **Texas**: Bandera Co., Myrtle Creek, FR 2828 S of Camp Verde, 29.8242, -99.1347, 3.IV.2004, B.C. Kondratieff and R.E. Zuellig, 4♂, 13♀ (CSUC); Little Creek, Farm Rd. 470 E of Utopia, 29.6514, -99.4767, 3.IV.2004, B.C. Kondratieff and R.E. Zuellig, ♂ (CSUC); Hays Co., Barton Creek, Hwy 12, N of Dripping Springs, 30.2380, -98.0665, 14.III.1993, B.C. Kondratieff and R.W. Baumann, 5♂, 21♀ (BYUC, CSUC); Paradise Hills, 25 January 1988, B.P. Stark, 2♂, 2♀ (BPSC); Travis Co., tributary of Barton Creek, near bowery of Hill Country Preserve in Bee Caves, downstream of Hwy 71, 20.III.1997, C.R. Nelson, 18♂, 13♀ (BYUC); Uvalde Co., Cherry Creek, Farm Rd. 1050 W of Utopia, 29.6061, -99.6925, 3.IV.2004, B.C. Kondratieff and R.E. Zuellig, 54♂, 38♀ (CSUC); Bear Creek, Farm Rd, 1050 W of Utopia, 29.5989, -99.5664, 3.IV.2004, B.C. Kondratieff and R.E. Zuellig, 72♂, 68♀ (CSUC).

##### Distribution.

USA: TX (DeWalt et al. 2012)

##### Remarks.

This species is somewhat superficially similar to *Zealeuctra fraxina*. Males are easily identified by the combination of the simple, U-shaped cleft and the presence of the anterior, concave cusp present at the base of the short, compact epiproct spine. The depiction of the posterior cusp as pointed and acute in Ricker and Ross (1969, their fig. 2) is not accurate and typically not visible. The cusp tends to be low and nub-like. This species is known mainly from the Edwards Plateau of west-central Texas (Fig. 12).

#### Zealeuctra cherokee

Stark & Stewart

http://species-id.net/wiki/Zealeuctra_cherokee

Figs 2 , 14

Zealeuctra cherokee Stark & Stewart, 1973: 192. Holotype ♂ (USNM), 2 mi W Vian, Sequoyah Co., Oklahoma

##### Description.

**Figures 2.**
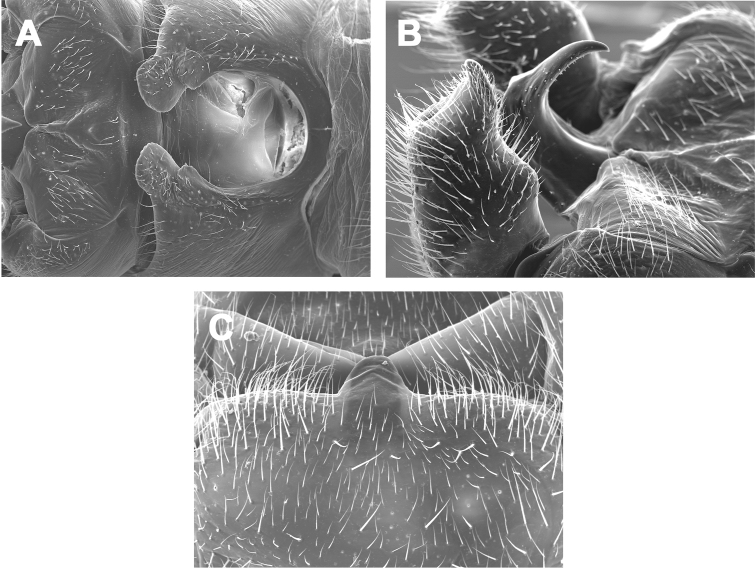
*Zealeuctra cherokee*, scanning election micrographs, USA, Oklahoma, Adair Co., 9 mi S Stillwell, Hwy 59, 20 February 1972. **A** male, cleft, dorsal view, 200× **B** male, abdominal terminalia, lateral view, 350× **C** female, posteromedial portion of seventh abdominal sternite, 350×.

**Male - abdominal tergal cleft.** Anterior portion U-shaped and very broadly rounded, posterior portion narrowing distally to a pair of medial projections, the terminal projection larger, subtruncate, and thumb-like, the subterminal projection smaller and subtriangular (Fig. 2A). Crenulations absent from inner margins of cleft.

**Male - epiproct.** Broad at base, extending posteriorly along one plane then extended abruptly and dorsally at a ca. 90 degree angle (Fig. 2B), spine tip slightly recurved anteriorly. No accessory spine or cusp present.

**Female - 7^th^ sternum.** Seventh sternum with a small, subtriangular lobe nested in a distinct central notch (Fig. 2C).

##### Material examined.

**USA**, **Arkansas:** Perry Co., Greathouse Creek, Hwy 216, 3 mi NE Thornburg, 34.9574, -92.7545, 7.IV.1984, B.C. Poulton, ♂ (CSUC); Sebastian Co., tributary to Sugar Creek, 5 mi SW Hartford, 17.II.1985, B.C. Poulton, ♂, ♀ (BYUC). **Oklahoma**: Adair Co., 9 mi S Stillwell, Hwy 59, 35.6917, -94.6691, 20.II.1972, B.P. Stark, 4♂, 5♀ (Paratypes, BPSC).

##### Distribution.

USA: AR, OK (DeWalt et al. 2012)

##### Remarks.

The cleft and epiproct spine of the male of this species are very similar to that of *Zealeuctra narfi*. The major difference for *Zealeuctra cherokee* is the presence of the posterior thickening along the recurved portion of the epiproct spine, and additionally, the subtruncate terminal medial processes at the posterior end of the cleft. The ranges of these two species broadly overlap in Arkansas and eastern Oklahoma (Fig. 14; Poulton and Stewart 1991).

#### Zealeuctra claasseni

(Frison)

http://species-id.net/wiki/Zealeuctra_claasseni

Figs 3 , 12

Leuctra claasseni Frison, 1929 (in part): 404. Holotype ♂ (INHS), Bushy Fork, Herod, Illinois.
Leuctra claasseni Frison, 1935 (in part): 354.
Leuctra claasseni Frison, 1942 (in part): 256.
Leuctra (Zealeuctra) claasseni Ricker, 1952: 173.
Zealeuctra claasseni Illies, 1966: 120.
Zealeuctra claasseni Ricker & Ross, 1969: 1115.

##### Description.

**Figures 3.**
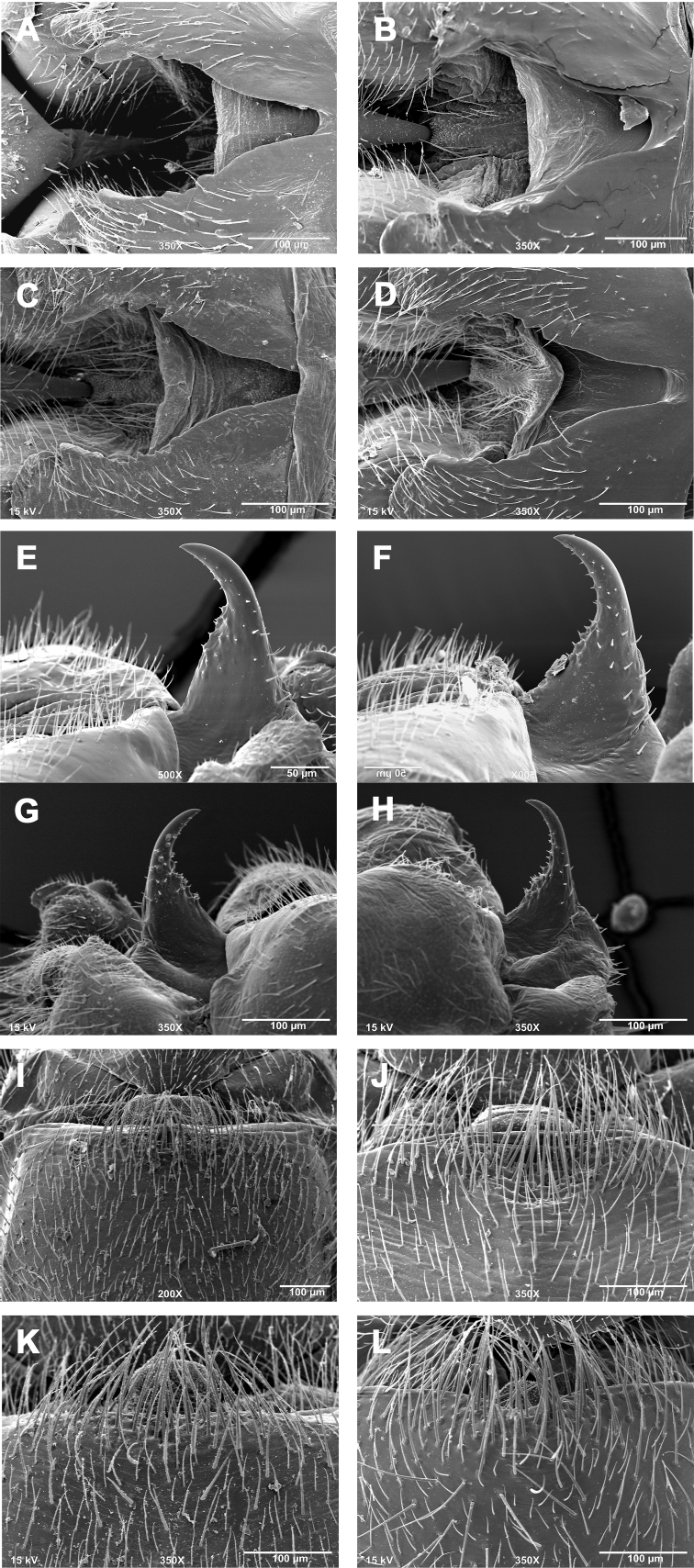
*Zealeuctra claasseni*, scanning electron micrographs, USA, Indiana, Franklin Co., Blue Creek, 20 March 2002 (**A, E, I**), Kentucky, Monroe Co., Little Sulphur Creek, 18 March 2001 (**B, F, J**), Missouri, Hog Creek, 17 March 2002 (**C, G, K**) Oklahoma, West Cache Creek, 5 February 2003 (**D, H, L**). **A–D** male, cleft, dorsal view, 350× **E–H** male, epiproct, lateral view, 350× or 500× **I–L** female, posteromedial portion of seventh abdominal sternite, 200× or 350×.

**Male - abdominal tergal cleft.** Anterior portion V-shaped with slight inward medial swelling but lacking crenulations along inner margins, anterior terminus very narrowly rounded (Figs 3A–D). Posterior portion slightly more U-shaped with several irregularly-sized and rounded teeth projecting medially.

**Male - epiproct.** Triangular base narrowing to anteriorly-recurved and tapering terminal spine, no accessory spine or swelling present (Figs 3E–H). Conspicuous tubercles located on the anterior margins of the triangular base. No accessory spine or cusp present.

**Female - 7^th^ sternum.** Seventh sternum with a small, variably-shaped lobe nested in a small central notch (Figs 3I–3L). The lobe ranges in shape from somewhat quadrate to broadly convex. The notch is likewise variably shaped, from essentially straight and scarcely perceptible (Figs 3I, 3K) to slightly concave (Figs 3J, 3L). Posterior margin essentially straight.

##### Material examined.

**USA**, **Arkansas:** Baxter Co., High Tower Creek, Hwy 126 S of Monkey Run, 36.3369, -92.4743, 16.III.2002, B.C. Kondratieff and R.E. Zuellig, 31♂, 24♀ (CSUC); Logan Co., West Fork Hegwood Creek, Hwy 22, 3 mi E Paris, 35.2950, -93.6782, 21.III.1984, B.C. Poulton, 2♂, 3♀ (BYUC); Newton Co., Buffalo River, Hwy 21 bridge S of Boxley, 35.9610, -93.4042, 10.III.2002, B.C. Kondratieff and R.E. Zuellig, 8♂, 3♀ (CSUC). **Illinois:** Alexander Co., tributary to Sandy Creek, 4.8 km WNW Tamms, 37.2450, -89.3210, 25.IV.2001, D.W. Webb, ♂, ♀ (INHS); Hardin Co., Threemile Creek, 6.5 km WNW Elizabethtown, 5.IV.2000, R. E. DeWalt, ♂, ♀ (INHS); Jackson Co., tributary to Big Muddy River, Clear Springs Picnic Area, 3.5 km ESE Howardton, 37.6234, -89.4255, 7.IV.1992, M.A. Harris and M.J. Wetzel, ♂, ♀ (INHS); Pope Co., Burden Branch, Burden Falls, Shawnee National Forest, 37.5633, -88.6424, 20.IV.1992, D.W. Webb and M.A. Harris, ♂, 3♀ (INHS); Dog Creek, 9 km NW Hamletsburg at CR 1, Shawnee National Forest, 37.2041, -88.4914, 10.III.2011, R.E. DeWalt and M.R. Jeffords, 17♂, 9♀ (INHS); Gibbons Creek, 0.8 km N Herod, 37.5842, -88.4422, 5.IV.2000, R.E. DeWalt, ♂ (INHS); tributary to Lusk Creek, 0.8 km N Rising Sun, 37.4156, -88.5797, 28.III.2006, R.E. DeWalt, 3♂, 6♀ (INHS); Lusk Creek, SE of Eddyville, 37.4729, -88.5472, 28.III.2006, R.E. DeWalt, ♂, 3♀ (INHS). **Indiana:** Brown Co., Jackson Creek, 8 km W Nashville, Yellowwood State Forest, 39.2075, -86.3461, 7.IV.2001, S.A. Grubbs, ♂, ♀ (WKUC); Spanker Branch, 14 km S Nashville, 39.0700, -86.2623, 7.IV.2001, S.A. Grubbs, ♂, 2♀ (WKUC); Skinner Creek, 8 km SSE Nashville, Brown County State Park, 39.1395, -86.2066, 7.IV.2001, S.A. Grubbs, 7♂, 6♀ (WKUC); Clark Co., Nine Penny Branch, 4 km NE Charleston, Nine Penny Branch Nature Preserve, 38.4772, -85.6318, 13.III.2000, S.A. Grubbs and J.M. Ferguson, 4♂ (WKUC); Crawford Co., small spring-fed stream, Rich Cave Hollow, 2.5 km N Branchville, Saalman Hollow Nature Preserve, 38.1907, -86.5732, 12.III.2000, S.A. Grubbs and J.M. Ferguson, ♂, 1 nymph (WKUC); Mitchell Creek, 2 km SE Birdseye, Hoosier National Forest, 38.3006, -86.6599, 23.III.2006, S.A. Grubbs and R.E. DeWalt, 19♂, 11♀ (WKUC, INHS); Little Blue River, Rte. 37, 3 km N Sulphur, Hoosier National Forest, 38.2521, -86.4782, 23.III.2006, S.A. Grubbs and R.E. DeWalt, ♂ (WKUC); Dearborn Co., tributary to East Fork Tanners Creek, 11 km E Sunman, 39.2364, -84.9684, 20.III.2002, S.A. Grubbs, ♂ (WKUC); Dubois Co., small spring-fed stream, 7 km NE Ferdinand, Ferdinand State Forest, 38.2584, -86.7897, 6.IV.2001, S.A. Grubbs, 5♂, 4♀, 2 nymphs (WKUC); Franklin Co., Blue Creek, 7 km SSW Brookville, 39.3572, -85.0351, 20.III2002, S.A. Grubbs, 11♂, 8♀ (WKUC); creek at Mt. Carmel, Hwy 252, 28.III.1953, A.R. Gaufin, 35♂, 25♀ (BYUC); Jackson Co., Guthrie Creek, 18 km E Bedford, Hemlock Bluff Nature Preserve, 38.8492, -86.2615, 14.III.2000, S.A. Grubbs and J.M. Ferguson, 13♂, 4♀ (WKUC); tributary to Little Salt Creek, 7 km WSW Waymansville, Hoosier National Forest, 39.0030, -86.1968, 7.IV.2001, S.A. Grubbs, 8♂, 3♀ (WKUC); Jefferson Co., Little Doe Run, 12 km W Vevay, Splinter Ridge Fish and Wildlife Area, 38.7483, -85.2239, 20.III.2002, S.A. Grubbs, 7♂, 7♀ (WKUC); Monroe Co., tributary to Clear Creek, 3 km NW Herrodsburg, Cedar Bluffs Nature Preserve, 39.0364, -86.5636, 24.III.2006, R.E. DeWalt and S.A. Grubbs, 5♂, 2♀ (INHS, WKUC); Montgomery Co., Indian Creek, 8 km N Waveland, Pine Hills Nature Preserve, 39.9421, -87.0503, 9.IV.2006, R.E. DeWalt, 2♂, 3♀ (INHS); Ohio Co., tributary to Willow Creek, 5 km NW Enterprise, 38.9151, -85.0232, 20.III.2002, S.A. Grubbs, 6♂, 3♀ (WKUC); Parke Co., Rocky Hollow Creek, 6 km NNW Marshall, Rocky Hollow Falls Canyon Nature Preserve, 39.8952, -87.1990, 9.IV.2006, R.E. DeWalt, ♂, ♀ (INHS); Perry Co., East Deer Creek, 13 km E Tell City, Hoosier National Forest, 37.9508, -86.6144, 12.III.2000, S.A. Grubbs and J.M. Ferguson, 11♂, 2♀ (WKUC); tributary to East Deer Creek, 13 km E Tell City, Hoosier National Forest, 37.9506, -86.6140, 12.III.2000, S.A. Grubbs and J.M. Ferguson, 2♂, 3♀, 5 nymphs (WKUC); Pike Co., tributary to Patoka River, Rte.257, 1 km E Velpen, 38.3576, -87.0914, 8.IV.2000, S.A. Grubbs, ♂, 7♀ (WKUC); Putnam Co., tributary to Big Walnut Creek, 3 km ESE Bainbridge, Hall Woods Nature Preserve, 39.7579, -86.7807, 20.IV.2008, R.E. DeWalt and S.K. Ferguson, ♂ (INHS); Spencer Co., tributary to Anderson River, 11 km N Troy, 38.0891, -86.8018, 6.IV.2001, S.A. Grubbs, 2♂, 3♀ (WKUC). **Kansas:** Douglas Co., temporary stream, T12S, R20, S4¸10.V.1983, D.G. Huggins, ♂, 2♀ (BYUC). **Kentucky:** Cumberland Co., Marrowbone Creek, Leatherwood Rd. nr. Rte. 90, 6 km W Marrowbone, 36.8361, -85.5648, 27.III.2013, S.A. Grubbs and J.M. Yates, ♂, 4♀ (WKUC); Edmonson Co., Cub Creek, Rte. 70, 4 km W Roundhill, 37.2421, -86.3873, 30.III.2013, S.A. Grubbs, ♂, 2♀ (WKUC); tributary to Cub Creek, Rte. 70, 37.2397, -86.3961, 30.III.2013, S.A. Grubbs, 16♂, 7♀ (WKUC); Chenneth Branch, Shrewsbury Rd., 37.3709, -86.3473, 30.III.2013, S.A. Grubbs, 3♂, 3♀ (WKUC); Grayson Co., Buck Creek, KY 79 bridge SW of Caneyville, 37.4058, -86.5109, 22.II.1999, B.C. Kondratieff and R.F. Kirchner, ♂, ♀ (CSUC); Hancock Co., tributary to North Branch South Fork Panther Creek, Rte. 1700, 10 km N Fordsville, 37.7246, -86.6737, 16.III.2002, S.A. Grubbs, ♂, 4♀ (WKUC); Marion Co., Sulfur Lick Creek, 6 km SE New Hope at Sulfur Lick Rd., 37.5876, -85.4993, 11.IV.2009, R.E. DeWalt and E.T. Chabot, ♂, 5♀ (INHS); Metcalfe Co., East Fork Little Barren River, Delk Branch Road, 12 km N Marrowbone, 36.9387, -85.5075, 27.III.2013, S.A. Grubbs and J.M. Yates, 2♂, ♀ (WKUC); tributary to East Fork Little Barren River, Reese Hurt Road, 12.5 km N Marrowbone, 36.9457, -85.5188, 27.III.2013, S.A. Grubbs and J.M. Yates, 9♂, 3♀ (WKUC); tributary to Marrowbone Creek, Rte. 90, Marrowbone State Forest, 36.8487, -85.6081, 29.III.2009, S.A. Grubbs, ♂ (WKUC); same but 27.III.2013, S.A. Grubbs and J.M. Yates, ♂, ♀ (WKUC); Moccasin Creek, Glen Shaw Rd., 10.5 km NW Summer Shade, 36.9164, -85.5917, 27.III.2013, S.A. Grubbs and J.M. Yates, 3♂, 3♀ (WKUC). Monroe Co., Little Sulphur Creek, Rte. 100/214 junction, 9.5 km E Tompkinsville, 36.7057, -85.5932, 18.III.2001, S.A. Grubbs, 11♂, 6♀, nymph (WKUC); Ohio Co., tributary to Pond Run, Rte. 110, 6 km W Falls of Rough, 37.5870, -86.6143, 16.III.2002, S.A. Grubbs, 6♂, 5♀ (WKUC); Trigg Co., Elbow Creek, Land-Between-The-Lakes, 36.7698, -88.0350, 18.III.2000, S.A. Grubbs, ♂ (WKUC); Warren Co., Doty Creek, Hays-Pondsville Rd., 11 km SW Bon Ayr, 36.9782, -86.1577, 24.III.2001, S.A. Grubbs, 3♂, 5♀ (WKUC); small temporary stream, Beckham Rd., 11.5 km SW Bon Ayr, 36.9648, -86.1745, 24.III.2001, S.A. Grubbs, ♂, ♀ (WKUC). **Missouri:** Barry Co., East Fork Rock Creek, Hwy M N of Mano, 36.5947, -93.6988, 16.III.2004, B.C. Kondratieff, R.E. Zuellig, and M. Garhart, 10♂, 3♀ (CSUC); tributary to Roaring River, CR 1162, Chute Ridge, 36.5612, -93.7936, 16.III.2004, B.C. Kondratieff, R.E. Zuellig, and M. Garhart, 12♂, 8♀ (CSUC); tributary to Rock Creek, Hwy M N of Mano, 36.6100, -93.7008, 16.III.2004, B.C. Kondratieff, R.E. Zuellig, and M. Garhart, 3♂, 3♀ (CSUC); Ozark Co., Lick Creek, Hwy J N of Howards Ridge, 36.5502, -92.3437, 16.III.2002, B.C. Kondratieff and R.E. Zuellig, 10♂, 4♀ (CSUC); Texas Co., West Fork Roubidoux Creek, Hwy M North of Huggins, 37.3534, -92.2091, 17.III.2002, B.C. Kondratieff and R.E. Zuellig, 38♂, 13♀ (CSUC); Hog Creek, Lily Rd. S of Houston, 37.2400, -91.9527, 17.III.2002, B.C. Kondratieff and R.E. Zuellig, 42♂, 15♀ (CSUC); Webster Co., Finley Creek, Hwy K S of Seymour, 37.1042, -92.7606, 17.III.2004, B.C. Kondratieff and R.E. Zuellig, 28♂, 15♀ (CSUC). **Ohio:** Clermont Co., stream, 2 mi W Neville, Rt. 52, 27.III.1974, O.S. Flint, 5♂, 6♀ (BYUC); Hocking Co., East Fork Salt Creek, Jct. OH 374/OH 56, 5.8 km ESE South Bloomingville, 39.3938, -82.5397, 18.IV.2010, R.E. DeWalt, ♂, 2♀ (INHS); Hoy Hollow Creek, 6.7 km E South Bloomingville, Hocking Hills State Park, 39.4236, -82.5221, 18.IV.2010, R.E. DeWalt, ♂, ♀ (INHS); East Fork, Ash Cave, 39.3954, -82.5473, 21.III.1975, R.W. Baumann and O.S. Flint, 4♂, ♀ (BYUC), same but 14.IV.1990, Clark and Wells, ♂, 2♀ (BYUC); Logan Co., tributary to Macochee Creek, 1 mi S Pickrelltown, 40.2785, -83.6741, 21.IV.1989, R.W. Baumann and R.F. Kirchner, ♂, ♀ (BYUC); Ross Co., Ralston Run, Hwy 772 and Blain Hwy, 39.2413, -83.0597, 19.IV.1989, R.W. Baumann and R.F. Kirchner, 3♂, 3♀ (BYUC); Crooked Creek, Jct. Blain Hwy and Mt Tabor Rd., 39.2140, -83.0293, 19.IV.1989, R.W. Baumann and R.F. Kirchner, 2♂ (BYUC). **Oklahoma:** Comanche Co., Cedar Creek, Wichita Mountain National Wildlife Reserve, 34.7248, -98.6739, 4.I.2010, R.E. DeWalt and S.K. Ferguson, 13♂, 3♀ (INHS); West Cache Creek, border Wichita NWR, Fort Sill, W Hwy 115, 34.6814, -98.6644, 5.II.2003, B.C. Kondratieff, R.E. Zuellig, and J.P. Schmidt, 12♂, 7♀ (CSUC); tributary to Blue Beaver Creek, Deer Creek Rd. at Blue Beaver Valley Rd., 34.7000, -98.5653, 15.III.2004, B.C. Kondratieff, R.E. Zuellig, and M. Garhart, 6♂, 4♀ (CSUC); Medicine Creek, Punch Bowl Rd., Fort Sill, 34.7169, -98.4903, 5.II.2003, B.C. Kondratieff, R.E. Zuellig, and J.P. Schmidt, 2♂, ♀ (CSUC); Blue Beaver Creek, McKenzie Hill Rd., Fort Sill, 34.6608, -98.5542, 5.II.2003, B.C. Kondratieff, R.E. Zuellig, and J.P. Schmidt, 25♂, 8♀ (CSUC); Johnston Co., Rock Creek, 5.8 km SE Mill Creek at OK 7, 34.3612, -96.7887, 3.I.2010, R.E. DeWalt and S.K. Ferguson, 4♂ (INHS); Latimer Co., Rock Creek, CR NE1130, 35.0186, -95.0599, 27.XII.2006, B.P. Stark, 3♂, 2♀ (BPSC).

##### Distribution.

USA: AL, AR, IL, IN, KS, KY, MO, OK, OH, TN, TX, WV (DeWalt et al. 2012)

##### Remarks.

Despite the broad distribution (Fig. 12), there appears to the little variation in the general features of the cleft. Only a minor degree of variation is evident in the epiproct, particular in the size and shape of the triangular base. Ricker and Ross (1969, their fig. 20) and Poulton and Stewart (1991, their fig. 112) depicted an epiproct base that is markedly broader than what is shown here with SEM micrographs.

#### Zealeuctra fraxina

Ricker & Ross

http://species-id.net/wiki/Zealeuctra_fraxina

Figs 4 , 11

Zealeuctra fraxina Ricker & Ross, 1969: 1117. Holotype ♂ (INHS), 5 mi S Hardinsburg, Breckinridge Co., Kentucky

##### Description.

**Figures 4.**
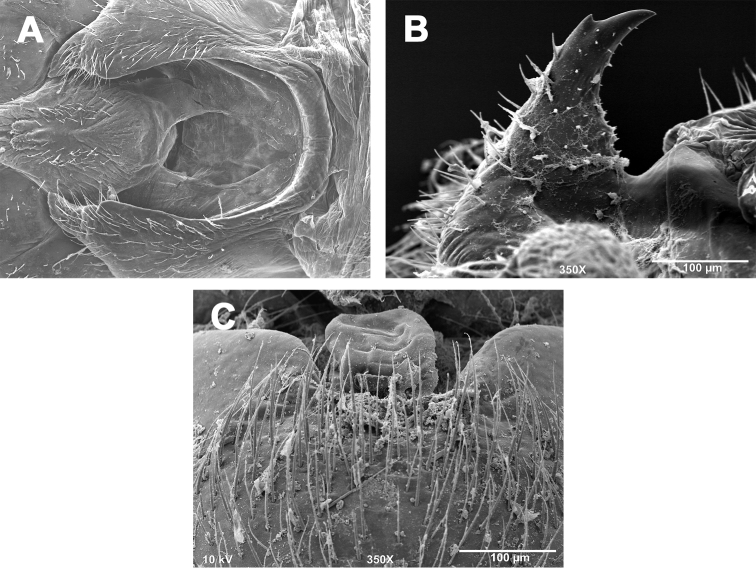
*Zealeuctra fraxina*, scanning electron micrographs, USA, Alabama, Jackson Co., tributary to Larkin Fork, Paint Rock River, 18 February 2006 (**A–B**), USA, Indiana, Martin Co., tributary to Lost River, 6 April 2001 (**C**). **A** male, cleft, dorsal view, 200× **B** male, epiproct, lateral view, 350× **C** female, posteromedial portion of seventh abdominal sternite, 350×.

**Male - abdominal tergal cleft.** Anterior portion wide and very broadly U-shaped, posterior portion also broadly rounded and bearing a slightly-concave serrated medial projection (Fig. 4A). Crenulations absent from inner margins of cleft.

**Male - epiproct.** Triangular base narrowing somewhat to an anteriorly-recurved and tapering terminal spine; a small accessory posterior spine is present but manifested only as a cusp-like projection on some specimens (Fig. 4B).

**Female - 7^th^ sternum.** Seventh sternum with a large, convex lobe nested in a distinct, central notch. The notch is bordered laterally by large, convex “shoulders” (Fig. 4C).

##### Material examined.

**USA**, **Alabama:** Jackson Co., tributary to Larkin Fork, Paint Rock River, Possum Hollow, Rte. 65, 1 km SE Francisco, 34.9852, -86.2421, 18.II.2006, S.A. Grubbs, 4♂, 12♀ (WKUC); Lawrence Co., tributary to West Fork Flint Creek, CR 56, 18 km NNW Addison, 34.3679, -87.1794, 7.II.2009, S.A. Grubbs, 2♂ (WKUC). **Illinois:** Saline Co., Battle Ford Creek, 3.5 km NE Delwood, 37.6050, -88.5440, 20.I.1993, D.W. Webb and M.A. Harris, ♂ (INHS). **Indiana:** Brown Co., Spanker Branch, 14 km S Nashville, 39.0700, -86.2623, 7.IV.2001, S.A. Grubbs, ♂, 4♀ (WKUC); Skinner Creek, 8 km SSE Nashville, Brown County State Park, 39.1395, -86.2066, 7.IV.2001, S.A. Grubbs, ♂ (WKUC); Crawford Co., small spring-fed stream, Rich Cave Hollow, Saalman Hollow Nature Preserve, 2.5 km N Branchville, 38.1907, -86.5732, 12.III.2000, S.A. Grubbs and J.M. Ferguson, 4♂, 4♀, 1 nymph (WKUC); tributary to Otter Creek, 1 km SE Taswell, Yellow Birches Ravine Nature Preserve, 38.3255, -86.5491, 14.III.2000, S.A. Grubbs and J.M. Ferguson, ♂ (WKUC); Floyd Co., tributary to Knob Creek, 17 km E Corydon, Brock-Sampson Nature Preserve, 38.1975, -85.9040, 13.III.2000, S.A. Grubbs and J.M. Ferguson, ♂, 3♀ (WKUC); Franklin Co., Salt Creek, 2 km W Peppertown at Bull Fork Rd., 39.4033, -85.2061, 4.II.2010, R.E. DeWalt and M. Pessino, 2♂, ♀ (INHS); West Fork Whitewater River, 1.4 km W Metamora at U.S. 52, 39.4507, -85.1495, 4.II.2010, R.E. DeWalt and M. Pessino, ♂, 3♀ (INHS); Harrison Co., tributary to Potato Run, 6 km E Leavenworth, Harrison-Crawford State Forest, 38.1876, -86.2766, 12.III.2000, S.A. Grubbs and J.M. Ferguson, 11♂, 12♀ (WKUC); Jackson Co., Combs Branch, 2.5 km NW Maumee at Tower Ridge Rd., 39.0316, -86.2832, 12.III.2010, M. Pessino, ♂, 3♀ (INHS). Lawrence Co., Sipes Branch, 4.5 km ENE Bartlettsville at Martin Hollow Rd, 38.9825, -86.3914, 12.III.2010, M. Pessino, 2♂, ♀ (INHS). Martin Co., tributary to Lost River, U.S. 150, 4 km SE Shoals, 38.6315, -86.7691, 6.IV.2001, S.A. Grubbs, 2♂, 9♀ (WKUC). Monroe Co., Allens Creek, 8 km SE Smithville, 39.0210, -86.4375, 14.III.2010, R.E. DeWalt and M. Pessino, ♂ (INHS). Orange Co., spring into Young’s Creek, 8 km S Paoli, 38.4903, - 86.4459, 20.II.2006, S.A. Grubbs, 2♂ (WKUC); Perry Co., East Deer Creek, 13 km E Tell City, Hoosier National Forest, 37.9508, -86.6144, 12.III.2000, S.A. Grubbs and J.M. Ferguson, 2♂, ♀ (WKUC); Scott Co., tributary to Big Ox Creek, 14 km SW Scottsburg, Clark State Forest, 38.5787, -85.8703, 15.III.2000, S.A. Grubbs and J.M. Ferguson, ♂ (WKUC). **Kentucky:** Cumberland Co., tributary to Bear Creek, Rte. 90, 15 km NW Albany, 36.7680, -85.2847, 18.III.2001, S.A. Grubbs, 6♂, ♀ (WKUC); Metcalfe Co., East Fork Little Barren River, Delk Branch Road, 12 km N Marrowbone, 36.9387, -85.5075, 27.III.2013, S.A. Grubbs and J.M. Yates, 2♂, ♀ (WKUC); tributary to East Fork Little Barren River, Reese Hurt Road, 12.5 km N Marrowbone, 36.9457, -85.5188, 27.III.2013, S.A. Grubbs and J.M. Yates, ♂, 3♀ (WKUC). **Ohio:** Lawrence Co., tributary to Storms Creek, 12 km SW Waterloo, Wayne National Forest, 38.6313, -82.5810, 26.II.2011, S.A. Grubbs, ♀ (WKUC). **Tennessee:** Sumner Co., tributary to Bledsoe Creek, Leaths Hollow Church Rd., 36.5153, -86.2437, 23.II.1998, B.C. Kondratieff and R.F. Kirchner, ♂ (CSUC); tributary to Little Trammel Creek, Sugar Grove, 36.6239, -86.2679, 22.II.1999, B.C. Kondratieff and R.F. Kirchner, 8♂, 8♀ (BPSC, BYUC); Little Trammel Creek, Rte. 174, nr. Sugar Grove, 36.6239, -86.2679, 18.I.2010, S.A. Grubbs, 2♂ (WKUC). **Virginia:** Bedford Co., Peaks of Otter Lake, 37.4454, -79.6029, 12.III.2013, E.M. Malloy, ♂ (WKUC).

##### Distribution.

USA: AL (Grubbs 2006), IL, IN, KY, NJ, OH, PA, TN, WV (DeWalt et al. 2012), VA (new state record)

##### Remarks.

Only *Zealeuctra claasseni* is distributed more broadly across the central and eastern US than *Zealeuctra fraxina* (Fig. 11; DeWalt et al. 2012). Additionally, this is one of only two species, *Zealeuctra talladega* being the other, that occur in the Appalachian Mountains. The Virginia record noted above was collected along the margins of Peaks of Otter Lake, but surprisingly several hundred meters from the nearest inlet or the only outlet (Little Stony Creek). *Zealeuctra fraxina* and *Zealeuctra arnoldi* are the only two species that bear a small subterminal cusp posterior to the tapering epiproct spine. Ricker and Ross (1969, their fig. 28) speculated that these two species, plus *Zealeuctra wachita*, share a common ancestor.

#### Zealeuctra hitei

Ricker & Ross

http://species-id.net/wiki/Zealeuctra_hitei

Figs 5 , 11

Zealeuctra hitei Ricker & Ross, 1969: 1118. Holotype ♂ (INHS), 3 mi S Johnson City, Blanco Co., Texas

##### Description.

**Figures 5.**
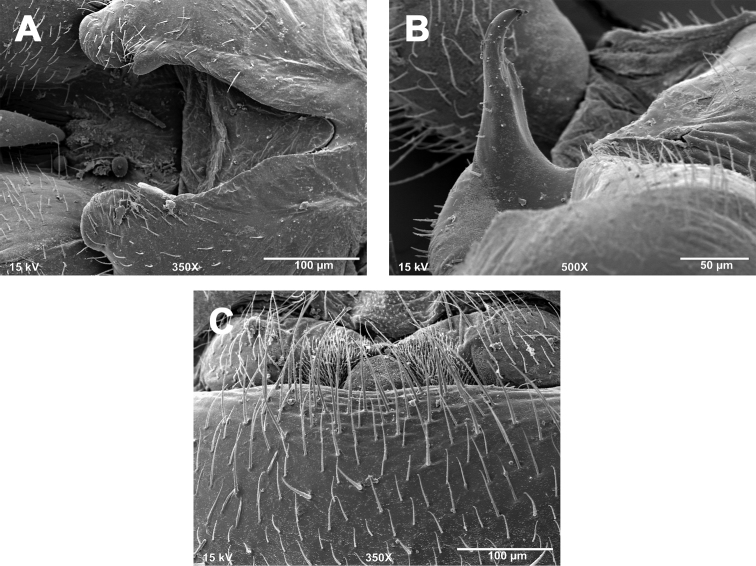
*Zealeuctra hitei*, scanning electron micrographs, USA, Texas, Kimble Co., Sycamore Creek, 14 December 1989. **A** male, cleft, dorsal view, 350× **B** male, epiproct, lateral view, 500× **C** female, posteromedial portion of seventh abdominal sternite, 350×.

**Male - abdominal tergal cleft.** Anterior portion nearly identical to *Zealeuctra claasseni*, with slight inward medial swelling but no apparent crenulations along inner margins (Fig. 5A). Posterior portion slightly more U-shaped with paired medially-projected processes, the terminal projection larger, convex, and thumb-like, the subterminal projection smaller and subtriangular.

**Male - epiproct.** Base slender and triangular, narrowing to anteriorly-recurved and tapering terminal spine, no accessory spine present (Fig. 5B). A small “step” (sensu Ricker and Ross 1969) demarks the base from the tapering spine. No accessory spine or cusp present.

**Female - 7^th^ sternum.** Seventh sternum with a small, subtriangular lobe nested in a scarcely-concave central notch (Fig. 5C). Posterior margin essentially straight.

##### Material examined.

**USA**, **Texas:** Coryell Co., Cowhouse Creek, Hwy 116 N of Copperas Cove, 31.2861, -97.8840, 21.XII.1969, K.W. Stewart, 2♂, ♀ (BPSC); Gillespie Co., stream at base of Summit Trail, Enchanted Rock State Natural Area, 30.4964, -98.8214, 19.I.1998, C.R. Nelson, 62♂, 23♀ (BYUC); Hays Co., Barton Creek, Hwy 12, N of Dripping Springs, 30.2380, -98.0665, 14.III.1993, R.W. Baumann and B.C. Kondratieff, ♂, ♀ (BYUC); small creek, 123 Rabbit Road, 11.IV.1992, S. Stringer, 2♂, ♀ (BYUC); Kimble Co., Sycamore Creek, Segovia, 30.4225, -99.6671, 14.XII.1989, B.C. Kondratieff and J.L. Welch, 3♂, 7♀ (CSUC); Travis Co., Barton Creek, Austin, near jct Lost Creek Blvd, 30.2739, -97.8449, 6.III.1997, C.R. Nelson, 2, 3 (BYUC).

##### Distribution.

USA: TX (DeWalt et al. 2012)

##### Remarks.

The form of the cleft of this species is nearly identical to *Zealeuctra claasseni*, and the epiproct is essentially a narrower form of that exhibited by *Zealeuctra claasseni*. Ricker and Ross (1969, their fig. 28) speculated that these two species share a common ancestor.

#### Zealeuctra narfi

Ricker & Ross

http://species-id.net/wiki/Zealeuctra_narfi

Fig. 6 , 14

Zealeuctra narfi Ricker & Ross, 1969: 1118. Holotype ♂ (INHS), Otter Creek, Sauk Co., Wisconsin

##### Description.

**Figures 6.**
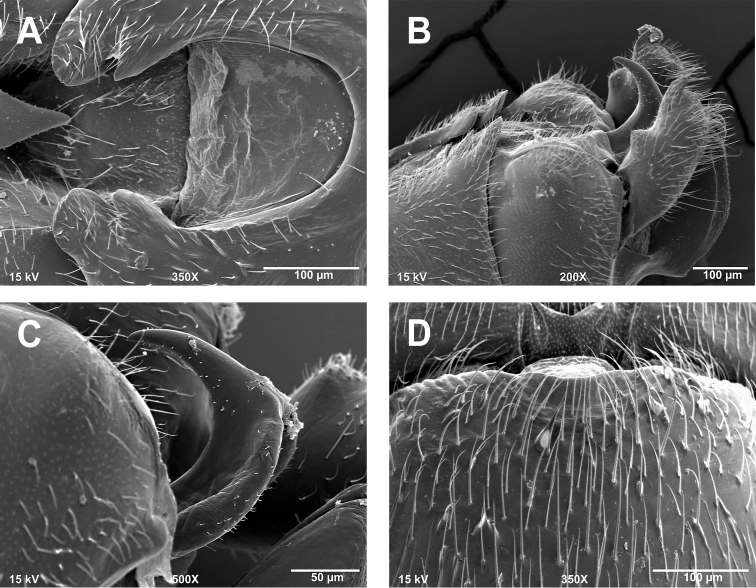
*Zealeuctra narfi*, scanning electron micrographs, USA, Missouri, Barry Co., East Fork Rock Creek, 16 March 2004. **A** male, cleft, dorsal view, 350×(**B** male, abdominal terminalia, lateral view, 200× **C** male, epiproct, lateral view, 500× **D** female, posteromedial portion of seventh abdominal sternite, 350×.

**Male - abdominal tergal cleft.** Anterior portion U-shaped and tapering slightly to a broadly-rounded anterior terminus (Fig. 6A). Posterior portion markedly narrower than anterior portion, with a pair of medially-projected processes (Figs 6A–B), the terminal projection larger, subtruncate, and thumb-like, the subterminal projection smaller and subtriangular.

**Male - epiproct.** Base broad and slightly-directed posterodorsally, tip of spine directed abruptly and anteriorly at ca. 90° angle from base, tapering and gently recurved anteriorly (Figs 6B–C). No accessory spine or cusp present.

**Female - 7^th^ sternum.** Seventh sternum with a broadly-subquadrate lobe nested in a scarcely-convex central notch (Fig. 6D). Posterior margins essentially straight.

##### Material examined.

**USA**, **Arkansas:** Pope Co., tributary to Little Creek, 4 mi NW Scottsville, 35.4776, -93.0836, 6.I.1985, B.C. Poulton, ♂ (BPSC); Sharp Co., unnamed creek, Cherokee Village, 36.2999, -91.5158, 4.III.1978, McGraw, 3♂ (CSUC); White Co., tributary to Little Cypress Creek, Hwy 5 at El Paso, 35.1264, -92.0974, 17.III.1984, B.C. Poulton, 2♂ (CSUC); Yell Co., 3 mi N Onyx, Hwy 27, 34.8899, -93.3937, 6.I.1999, B.P. Stark 2♂, 2♀ (BPSC). **Illinois:** Pope Co., tributary to Burden Branch, below Burden Falls, Shawnee National Forest, 37.5641, -88.6434, 3.IV.2013, S.A. Grubbs and J.M. Yates, 4♂, 12♀ (WKUC); tributary to Burden Branch, Shawnee National Forest, 37.5641, -88.6387, 3.IV.2013, S.A. Grubbs and J.M. Yates, 9♂, 12♀ (WKUC); Saline Co., Battle Ford Creek, 3.5 km NE Delwood, 37.6050, -88.5440, 26.II.1992, D.W. Webb and M.A. Harris, ♂ (INHS). **Missouri:** Barry Co., tributary to Rock Creek, Hwy M N of Mano (Big M), 36.6100, -93.7008, 16.III.2004, B.C. Kondratieff, R.E. Zuellig, and M. Garhart, 10♂, 8♀ (BYUC, CSUC); tributary to Roaring River, CR 1162, Chute Ridge, 36.5612, -93.7936, 16.III.2004, B.C. Kondratieff, R.E. Zuellig, and M. Garhart, 28♂, 13♀ (CSUC); East Fork Rock Creek, Hwy M N Mano, 36.5947, -93.6988, 16.III.2004, B.C. Kondratieff, R.E. Zuellig, and M. Garhart, 46♂, 19♀ (CSUC); Stone Co., creek with old dam, Million Oaks sub tract, Table Rock Reservoir, 17.III.1993, S. Fitzgerald, 35♂, 9♀ (CSUC).

##### Distribution.

USA: AR, IL, MO, OK, WI (DeWalt et al. 2012)

##### Remarks.

Although the type locality for *Zealeuctra narfi* is in southern Wisconsin, this species is common and widespread only in the southern portion of its range, and particularly in southern Missouri (Fig. 14). There are only three known locations for *Zealeuctra narfi* in Wisconsin (DeWalt unpublished data) and is likewise uncommon in Illinois, with only three collected localities between 1976 and 2000 (Webb 2002). There have also been several failed attempts by the senior author to locate this species in western and southern Indiana.

#### Zealeuctra stewarti

Kondratieff & Zuellig

http://species-id.net/wiki/Zealeuctra_stewarti

Fig. 13

Zealeuctra stewarti Kondratieff & Zuellig, 2004: 840. Holotype ♂ (TAMU), 5.2 mi E Leakey, Real Co., Texas

##### Description.

**Male - abdominal tergal cleft.** Anterior portion broadly U-shaped and parallel-sided. Posterior portion V-shaped, with small crenulations evident along inner margins, terminating posteriorly with paired, subtriangular, medially-projected extensions (Kondratieff and Zuellig 2004, their Fig. 1)

**Male - epiproct.** Epiproct spine long, slender, and gently-recurved anteriorly (Kondratieff and Zuellig 2004; their fig. 2). No accessory spine or cusp present.

**Female - 7^th^ sternum.** Seventh sternum lacking medial lobe, with posteromedial portion overlapping as a slightly-notched, subtruncate flap onto anteromedial margin of the eighth sternum Kondratieff and Zuellig 2004; their fig. 3).

##### Material examined.

**USA**, **Texas:** Real Co., Little Dry Frio, Farm Rd. 337 E of Leakey, 29.7214, -99.6739, 3.IV.2004, B.C. Kondratieff and R.E. Zuellig, 4♂, 6♀ (Paratypes; BYUC, CSUC); Little Dry Frio, Hwy 337, 5.2 mi E of Leakey, 29.7214, -99.6739, 20.II.2010, K.W. Stewart, 2♂ (BPSC).

##### Distribution.

USA: TX (DeWalt et al. 2012)

##### Remarks.

This is easily the rarest of the *Zealeuctra* species, currently known only from two streams within a very small geographic area in the Texas Hill Country region (Fig. 13; Kondratieff and Zuellig 2004), overlapping in range and adult flight periods only with *Zealeuctra arnoldi*. The long and very slender epiproct spine exhibited by *Zealeuctra stewarti* is unique and easily distinguished from all members of this genus. In addition, the females of *Zealeuctra stewarti* and *Zealeuctra warreni* are the only two *Zealeuctra* species lacking a posterior lobe nested in central notch and where the posteromedial portion of the 7^th^ sternum is flap-like and extending over the anteromedial margin of the 8^th^ sternum.

#### Zealeuctra talladega

Grubbs

http://species-id.net/wiki/Zealeuctra_talladega

Figs 7 , 13

Zealeuctra talladega Grubbs, 2005: 40. Holotype ♂ (INHS), tributary to Barbaree Creek, 22 km E Talladega, Clay Co., Alabama

##### Description.

**Figures 7.**
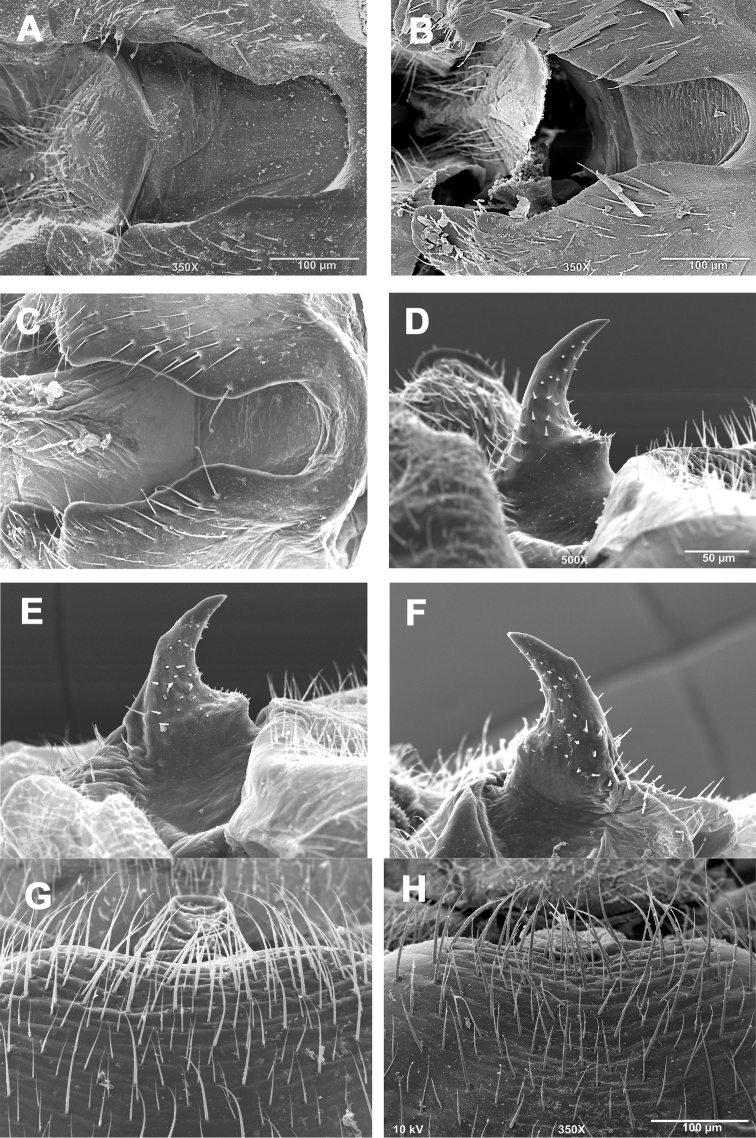
*Zealeuctra talladega*, scanning electron micrographs, USA, Alabama, Clay Co., tributary to Swept Creek, 24 January 2006 (**A–B, D–E, G**), USA, Alabama, Clay Co., tributary to West Fork Hatchet Creek, 25 January 2006 (**C, F, H**). **A–C** male, cleft, dorsal view, 350× **D–F** male, epiproct, lateral view, 500× **G–H** female, posteromedial portion of seventh abdominal sternite, 350×.

**Figures 8.**
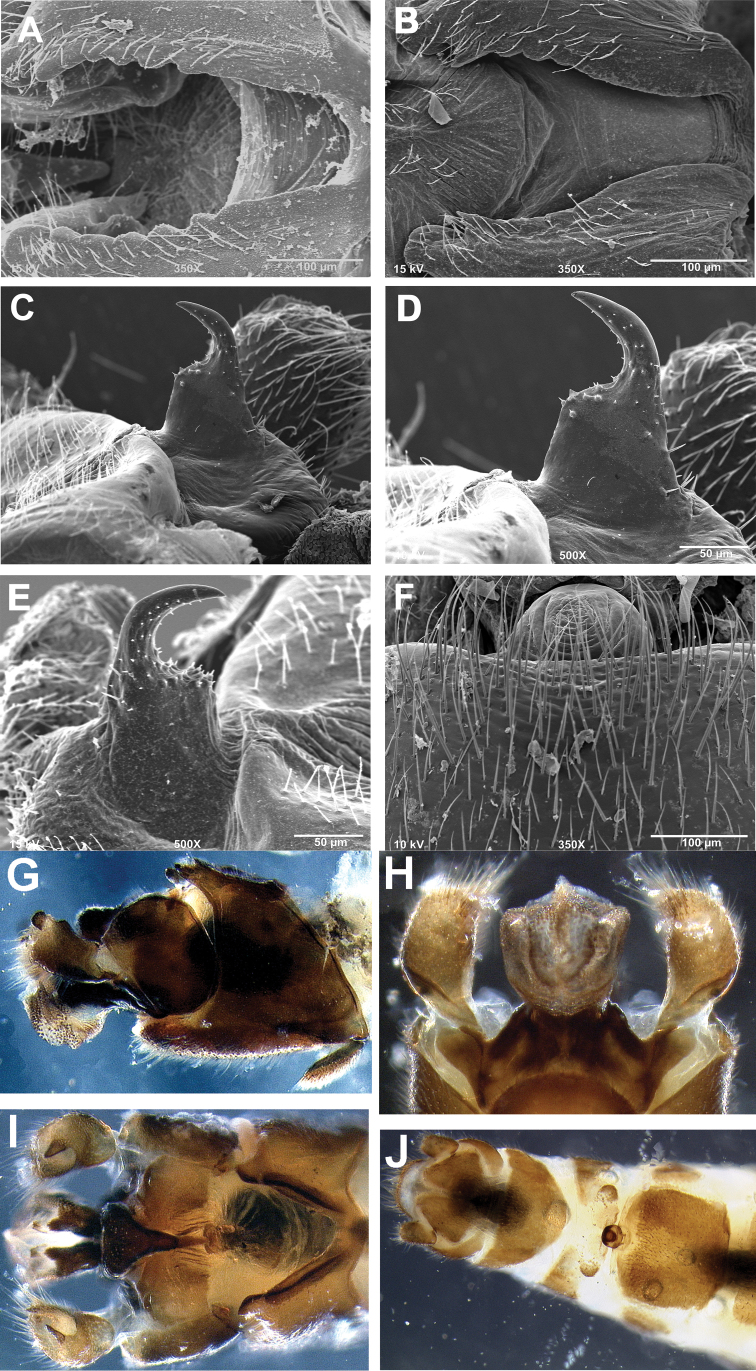
*Zealeuctra ukayodi*, sp. n., scanning electron micrographs, USA, Alabama, Jackson Co., Poplar Spring, 16 March 2008 (**A–D, F–J**), USA, Tennessee, Grundy Co., tributary to Elk River, 12 February 2007 (**E**). **A–B** male, cleft, dorsal view, 200× **C–E** male, epiproct, lateral view, 350× or 500× **F** female, posteromedial portion of seventh abdominal sternite, 350× **G** male terminalia, lateral **H** male terminalia, dorsal **I** male terminalia, ventral **J** female terminalia, ventral.

**Male - abdominal tergal cleft.** Anterior portion is near parallel-sided, U-shaped, and broadly rounded along anterior margin (Figs 7A–C). Posterior portion is somewhat V-shaped and sinuous along inner margins, posterior terminus marked by either a single (Fig. 7C) or paired (Figs 7A–8B) medially-directed subtriangular projections. Crenulations absent from inner margins of cleft..

**Male - epiproct.** Base very broad and subquadrate in shape, narrowing to anteriorly-recurved and broadly tapering terminal spine, no accessory spine present although a small subterminal posterior nub may be present (Figs 7D–F). Subquadrate base varies from rounded broadly to right angular in shape. No accessory spine or cusp present, but a minute, very low, medially-positioned hump is present along the anterior, recurved portion of epiproct (Figs 7D–F).

**Female - 7^th^ sternum.** Seventh sternum with a small, lobe nested in a scarcely-convex central notch. Lobe ranges in shape from subtriangular (Fig. 7G) to convex (Fig. 7H). Posterior margin moderately convex.

##### Material examined.

**USA**, **Alabama:** Clay Co., tributary to Barbaree Creek, 22 km E Talladega, Talladega National Forest, 33.4187, -85.8706, 16.II.2003, S.A. Grubbs and D.K. King, 2♂, 2♀ (WKUC – paratypes); same but 5.III.2012, S.A. Grubbs, ♂, 4♀ (WKUC); Swept Creek, Talladega National Forest, 33.2602, -86.1006, 23.I.2006, A.L. Sheldon, ♀ (WKUC); tributary to Swept Creek, Talladega National Forest, 33.2595, -86.1031, 23.I.2006, A.L. Sheldon, 3♂, 6♀ (WKUC); tributary to Swept Creek, Talladega National Forest, 33.2632, -86.0922, 24.I.2006, A.L. Sheldon, 7♂, 9♀ (WKUC); South Branch Swept Creek, Talladega National Forest, 33.2607, -86.0952, 24.I.2006, A.L. Sheldon, 2♂, 6♀ (WKUC); tributary to Swept Creek, Talladega National Forest, 33.2614, -86.0955, 24.I.2006, A.L. Sheldon, ♀ (WKUC); tributary to West Fork Hatchet Creek, Talladega National Forest, Forest, 33.2938, -86.0780, 25.I.2006, A.L. Sheldon, ♂ (WKUC); tributary to West Fork Hatchet Creek, Talladega National Forest, Forest, 33.2740, -86.0733, 25.I.2006, A.L. Sheldon, ♂, ♀ (WKUC); tributary to West Fork Hatchet Creek, Talladega National Forest, Forest, 33.2823, -86.0666, 25.I.2006, A.L. Sheldon, 4♂, 4♀ (WKUC); tributary to West Fork Hatchet Creek, Talladega National Forest, 33.2825, -86.0668, 8.III.2007, A.L. Sheldon, 3♀ (WKUC); tributary to West Fork Hatchet Creek, Talladega National Forest, 33.2743, -86.0739, 8.III.2007, A.L. Sheldon, 2♀ (WKUC); tributary to West Fork Hatchet Creek, Talladega National Forest, 33.3219, -86.0671, 7.IV.2008, A.L. Sheldon, 2♀ (WKUC); tributary to West Fork Hatchet Creek, FR 662, 16 km SSE Talladega, Talladega National Forest, 33.3048, -86.0380, 5.III.2012, S.A. Grubbs, ♂, 2♀ (WKUC); tributary to Hatchet Creek, FR 687, Talladega National Forest, 33.1557, -86.1196, 5.III.2012, S.A. Grubbs, 4♀ (WKUC); tributary to Cheaha Creek, Talladega National Forest, 33.4397, -85.8387, 5.III.2012, S.A. Grubbs, ♀ (WKUC); tributary to Tallaseehatchie Creek, FR 616, Talladega National Forest, 33.2053, -86.0800, 5.III.2012, S.A. Grubbs, ♂, 2♀ (WKUC); Cleburne Co., Cheaha Creek, above Cheaha Lake, Cheaha State Park, 2.III.1991, R.W. Baumann and S.M. Clark, 2♂, 2♀ (BYUC); Talladega Co., tributary to Smelley Creek, Talladega National Forest, 33.2988, -86.0842, 8.III.2007, A.L. Sheldon, ♂, 6♀ (WKUC), Smelley Creek, Talladega National Forest, 33.3010, -86.0945, 21.XII.2007, A.L. Sheldon, ♂ (WKUC); tributary to Smelley Creek, Talladega National Forest, 33.3006, -86.0862, 7.IV.2008, A.L. Sheldon, ♂, 2♀ (WKUC).

##### Distribution.

USA: AL (DeWalt et al. 2012)

##### Remarks.

This species is known only from the southern Talladega Mountains region of eastern Alabama (Fig. 13), the southern terminus of the Appalachian Mountains. Extensive collecting efforts by both the author and Dr. Andrew Sheldon since 2005 have yet to produce localities north from the two counties (Clay and Talladega) where this species has been obtained.

#### Zealeuctra ukayodi

Grubbs sp. n.

http://zoobank.org/7D5A6F56-AA11-458D-868B-CB7654BFD8C8

http://species-id.net/wiki/Zealeuctra_ukayodi

Figs 8 , 13

##### Description.

**Male.** Forewing length 6.5–7.0 mm; body length 5.0–5.5 mm. General body color brown.

Anterior portion of male abdominal tergal cleft is narrowly U-shaped, and rounded along the anterior terminus. The posterior portion is widest anteriorly with crenulations present along inner margins. Overall shape of cleft ranges from either V-shaped to somewhat sinuous (Figs 8A–B, I). Epiproct base very broad and subquadrate in shape, narrowing to anteriorly-recurved and broadly tapering terminal spine, no accessory spine present (Figs 8C–E). Subquadrate base varies in from rounded broadly to right angular in shape. No accessory spine or cusp present. Cerci sclerotized mainly along outer margin and bearing a small dorsomedial hump and a subapical, triangular sclerotized tooth (Figs 8G–I). Length of vesicle ca. 1.5× width.

**Female.** Forewing length 7.5–8.0 mm; body length 6.0–8.0 mm. General body color brown. Seventh sternum with a quadrate sclerotized region, convex posteriorly, and scarcely projecting over the anterior portion of the eighth sternum (Fig. 8I); posteromedial portion unpigmented and very slightly notched, bearing a lightly-pigmented lobe that is convex posteriorly (Figs 8F, I).

**Nymph.** Unknown.

##### Material examined.

Holotype ♂, in 95% ethyl alcohol, **USA**, **Alabama**, Jackson Co., Poplar Spring, 6 km SW Hytop, 34.8779, -86.1283, 19.II.2007, S.A. Grubbs (INHS). Paratypes: same as Holotype, 19.II.2007, S.A. Grubbs, 4♂, 7♀ (WKUC); same as Holotype but 16.III.2008, S.A. Grubbs, 13♂, 30♀ (INHS, WKUC). **Tennessee:** Cumberland Co., North Fork Elmore Creek, TN Rte. 298, 36.1037, -84.9414, 9.II.1998, B.C. Kondratieff and R.F. Kirchner, 2♂, 4♀ (CSUC); Grundy Co., tributary to Elk River, Rte. 50, 14 km N Monteagle, 35.3578, -85.8363, 12.II.2007, S.A. Grubbs, ♂, 2♀ (WKUC); Marion Co., tributary to Cross Creek, 17 km NW South Pittsburg, Franklin-Marion State Forest, 35.0847, -85.8673, 12.II.2007, S.A. Grubbs, ♂ (WKUC); tributary to Sweeten Creek, 15 km NW South Pittsburg, Franklin-Marion State Forest, 35.0827, -85.8391, 12.II.2007, S.A. Grubbs, 2♀ (WKUC); tributary to Sweeten Creek, 15 km NW South Pittsburg, Franklin-Marion State Forest, 35.0942, -85.8600, 8.II.2013, S.A. Grubbs, 3♂, 17♀ (WKUC); Cave Springs Creek, Franklin-Marion State Forest, 35.0764, -85.8427, 25.II.2007, A.L. Sheldon, ♂, ♀ (WKUC).

##### Etymology.

The specific epithet is a Cherokee word for “dry”, a figurative reference to the temporary stream habitat characteristic of this species. Cherokee Native Americans formerly inhabited the southern Cumberland Plateau region.

##### Diagnosis.

The species is similar only to the cognate *Zealeuctra talladega*, and these two species can be separated mainly by characteristics of the male cleft. In *Zealeuctra talladega*, the cleft is highly sinuous or hourglass in shape and lacks the large crenulations along the inner margins. In *Zealeuctra ukayodi*, the cleft ranges from broadly V-shaped to somewhat sinuous, with large, conspicuous crenulations present along the inner margins of the posterior portion. Variation in the shape of the epiproct, namely the anterior quadrate or subquadrate shelf, overlaps between the two species. There is also a minute, medially-positioned hump present along the anterior, recurved portion of epiproct in *Zealeuctra talladega* (Figs 7D–F) that is lacking entirely in *Zealeuctra ukayodi* (Figs 8C–E). The fused subanal plates-anal probe of *Zealeuctra talladega* and *Zealeuctra ukayodi* appears to be highly similar in structure.

##### Remarks.

*Zealeuctra ukayodi* appears to be restricted to the southern portion of the Cumberland Plateau, known at present from central Tennessee south to the type locality in northeastern Alabama (Fig. 13). The range of only one other *Zealeuctra* species, *Zealeuctra fraxina*, extends south and eastward into the Cumberland Plateau region in northeastern Alabama (Grubbs 2006). No other stonefly species have been collected at the type locality. *Allocapnia unzickeri* Ross & Yamamoto, *Oemopteryx contorta* (Needham & Claassen), and *Taeniopteryx ugola* Ricker & Ross have been obtained with *Zealeuctra ukayodi* at some of the paratype localities in Tennessee. The common name, Cumberland Needlefly, is proposed for this species (Stark et al. 2012).

#### Zealeuctra wachita

Ricker & Ross

http://species-id.net/wiki/Zealeuctra_wachita

Figs 9 , 11

Zealeuctra wachita Ricker & Ross, 1969: 1119. Holotype ♂ (INHS), Ouachita River, Polk Co., Arkansas

##### Description.

**Figures 9.**
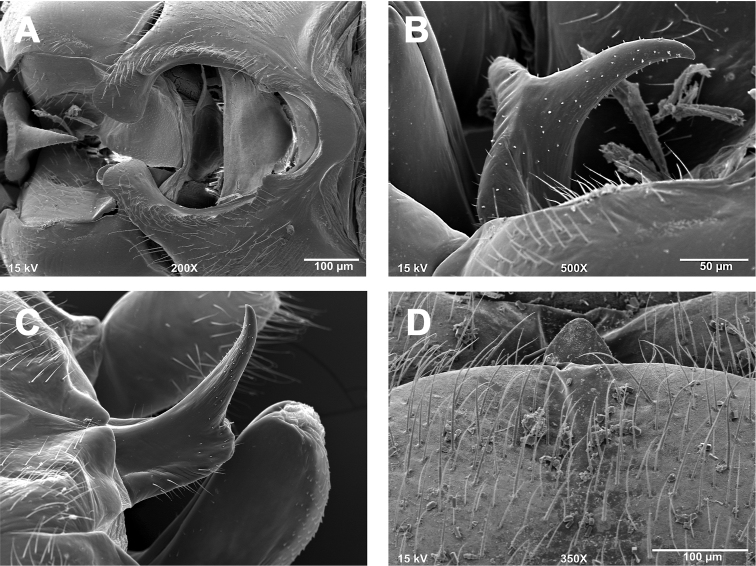
*Zealeuctra wachita*, scanning electron micrographs, USA, Oklahoma, LeFlore Co., tributary to Big Creek, 6 February 2003 (**A, B, D**), USA, Arkansas, Scott Co., Johnson Creek, 6 January 1999 (**C**). **A** male, cleft, dorsal view, 200× **B–C** male, epiproct, lateral view, 350× or 500× **D** female, posteromedial portion of seventh abdominal sternite, 350×.

**Male - abdominal tergal cleft.** Anterior portion broadly rounded, with a secondary U-shaped extension, inner margins set apart from remainder of cleft (Fig. 9A). Posterior portion markedly wider than anterior portion, interior margins rounded and lacking crenulations, terminating posteriorly as paired, large subtruncate medially-projected extensions.

**Male - epiproct.** Base narrow and extending laterally (Fig. 9A), spine directed at ca. 90° angle from base, tapering gradually, and recurved somewhat anteriorly (Figs 9B–C). No accessory spine or cusp present.

**Female - 7^th^ sternum.** Seventh sternum with a small, subtriangular lobe nested in a slightly-concave central notch (Fig. 9D).

##### Material examined.

**USA**, **Arkansas:** Scott Co., Johnson Creek, Hwy 49, 34.7131, -94.2101, 6.I.1999, B.P. Stark, 2♂, ♀ (BPSC). **Oklahoma:** LeFlore Co., tributary to Big Creek, NW Page, Hwy 59/270, 34.7144, -94.5547, 6.II.2003, B.C. Kondratieff, J.P. Schmidt, and R.E. Zuellig, 3♂, ♀ (CSUC); tributary to Big Creek, NW Page, Hwy 59/270, 34.7194, -94.5608, 6.II.2003, B.C. Kondratieff, J.P. Schmidt, and R.E. Zuellig, 4♂, 3♀ (CSUC); Big Creek, NW Page at Page Cemetery Rd., 34.7139, -94.5500, 6.II.2003, B.C. Kondratieff, J.P. Schmidt, and R.E. Zuellig, ♂ (CSUC); intermittent stream just E of Muse, Hwy 63, 34.6718, -94.7585, 15.III.2002, B.C. Kondratieff and R.E. Zuellig, 2♂ (CSUC).

##### Distribution.

USA: AR (DeWalt et al. 2012), OK (new state record)

##### Remarks.

This is the only *Zealeuctra* species that has a cleft bearing a secondary anterior extension. *Zealeuctra wachita* appears to be easily the least common of the three *Zealeuctra* species endemic to the Interior Plateau region. Poulton and Stewart (1991), in their study of the stoneflies of the Ozark and Ouachita Mountains, included only two Arkansas localities for this species. In contrast, the other two regional species, *Zealeuctra cherokee* and *Zealeuctra warreni*, plus *Zealeuctra claasseni* and *Zealeuctra narfi*, are markedly more common. The Oklahoma records noted above (Fig. 11) represent new state records.

#### Zealeuctra warreni

Ricker & Ross

http://species-id.net/wiki/Zealeuctra_warreni

Figs 10 , 13

Zealeuctra warreni Ricker & Ross, 1969: 1120. Holotype ♂ (INHS), Sugar Creek, 5 mi E Hardy, Sharp Co., Arkansas

##### Description.

**Figures 10.**
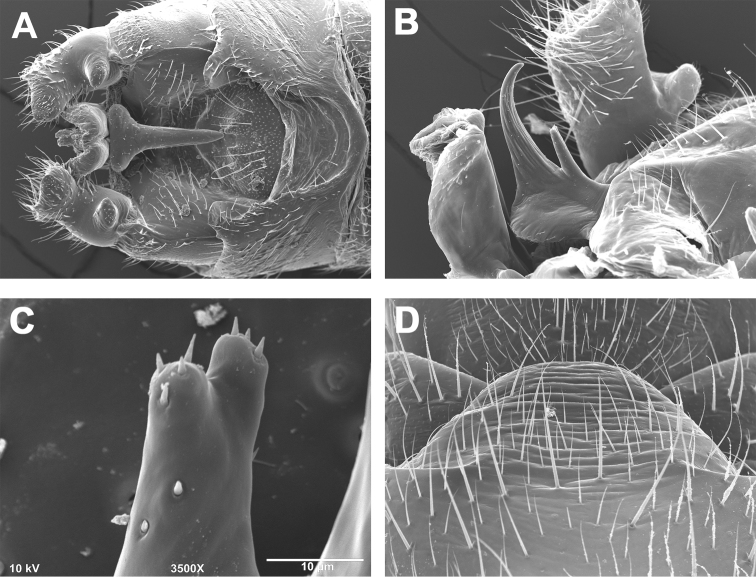
*Zealeuctra warreni*, scanning electron micrographs, USA, Arkansas, Polk Co., Rock Creek, 26 November 1983 (**A**), USA, Arkansas, Scott Co., Johnson Creek, 6 January 1999 (**B–D**). **A** male, cleft, dorsal view, 200× **B** male, epiproct, lateral view, 350× **C** male, tip of anterior accessory spine, anterior view, 3500× **D** female, posteromedial portion of seventh abdominal sternite, 350×.

**Male - abdominal tergal cleft.** Anterior portion broadly-rounded and U-shaped. Posterior portion V-shaped, with small crenulations evident along inner margins, terminating posteriorly with paired, subtriangular, medially-projected extensions (Fig. 10A).

**Male - epiproct.** Base broad and flanged laterally (Fig. 10A), with paired spines both originating from base, posterior spine ca. 2× length of anterior spine, moderately recurved anteriorly (Fig. 10B), anterior spine straight and bifurcated slightly at tip, with the paired terminal ends bearing four thick spines at tip (Fig. 10C).

**Female - 7^th^ sternum.** Seventh sternum lacking medial lobe, with posteromedial portion overlapping as a broadly-triangular flap onto anteromedial margin of the eighth sternum (Fig. 10D).

##### Material examined.

**USA**, **Arkansas:** Polk Co., Rock Creek, Hwy 71, 2 mi SW Mena, 34.5601, -94.2902, 26.X.1983, B.C. Poulton, 4♂ (CSUC); tributary to Casatot (sic Cossatot) River, 1.5 mi S Shady off AR 375, 34.4373, -94.1281, 11.XI.1990, S.R. Moulton & K.W. Stewart, 22♂, 19♀ (BYUC); Scott Co., Johnson Creek, Hwy 49, 34.7131, -94.2101, 6.I.1999, B.P. Stark, 2♂, 5♀ (BPSC); Van Buren Co., Archy Creek, S of Woolum, W of Botkinburg, 35.6883, -92.6500, 7.II.2003, B.C. Kondratieff, R.E. Zuellig and J.P. Schmidt, 2♂, 2♀ (CSUC); Washington Co., Wildcat Creek, CR 870, 36.1223, -94.2460, 17.I.1999, B.P. Stark ♂ (BPSC); West Fork of the White River, 0.5 mi N Brentwood, at rest stop, 35.8663, -94.1188, 25.XI.1995, C.R. Nelson, ♂, 4♀ (BYUC); Cove Creek, 15 mi S Prairie Grove, 35.7758, -94.3748, 17.XI.1962, O. Hite and M. Hite, 4♂, 8♀ (BYUC). **Oklahoma:** LeFlore Co., Big Creek, Page, 34.7160, -94.5503, 5.I.2006, B.P. Stark, 2♂, ♀ (BPSC).

##### Distribution.

USA: AR, MO, OK (DeWalt et al. 2012)

##### Remarks.

*Zealeuctra warreni* males are easily distinguished from all *Zealeuctra* species by presence of the two prominent epiproct spines. The common name for this species, Early Needlefly (Stark et al. 2012), is apt since its November–February emergence period is earlier compared to each of the four other regional congeners (*Zealeuctra cherokee*, *Zealeuctra claasseni*, *Zealeuctra narfi*, and *Zealeuctra wachita*) in the Interior Highlands. Poulton and Stewart (1991) noted the emergence period for *Zealeuctra warreni* starts in November.

**Figure 11.**
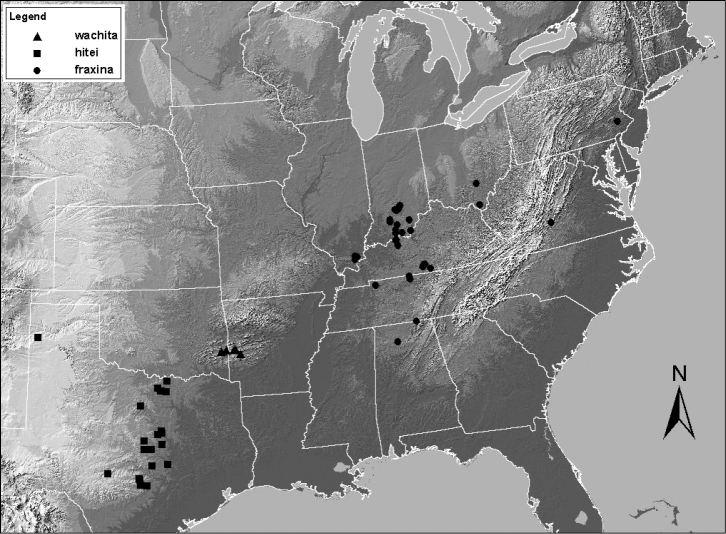
Distribution map for *Zealeuctra fraxina*, *Zealeuctra hitei*, and *Zealeuctra wachita*.

**Figure 12.**
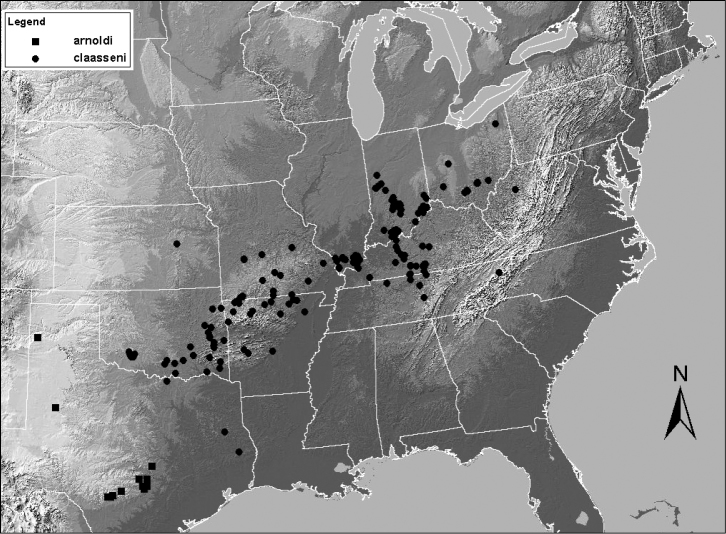
Distribution map for *Zealeuctra arnoldi* and *Zealeuctra claasseni*.

**Figure 13.**
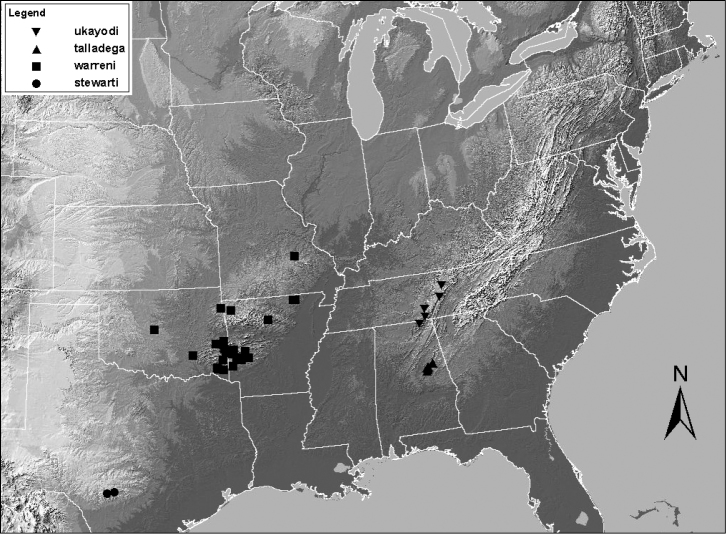
Distribution map for *Zealeuctra stewarti*, *Zealeuctra talladega*, *Zealeuctra ukayodi* sp. n., and *Zealeuctra warreni*.

**Figure 14.**
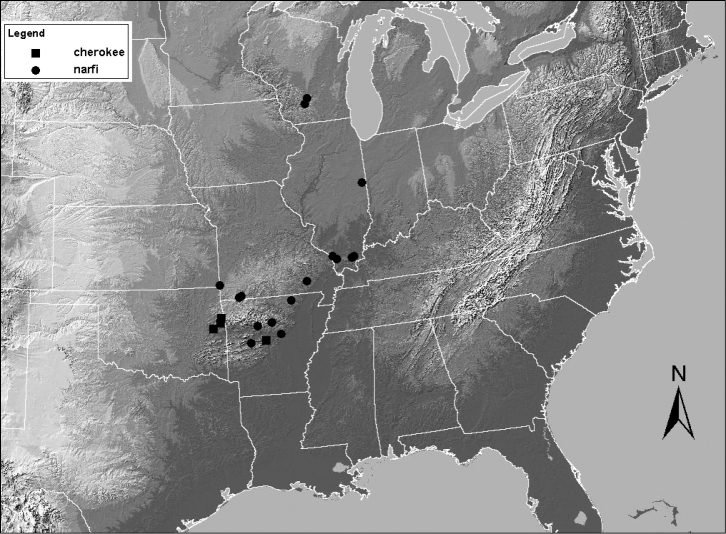
Distribution map for *Zealeuctra cherokee* and *Zealeuctra narfi*.

## Supplementary Material

XML Treatment for Zealeuctra arnoldi

XML Treatment for Zealeuctra cherokee

XML Treatment for Zealeuctra claasseni

XML Treatment for Zealeuctra fraxina

XML Treatment for Zealeuctra hitei

XML Treatment for Zealeuctra narfi

XML Treatment for Zealeuctra stewarti

XML Treatment for Zealeuctra talladega

XML Treatment for Zealeuctra ukayodi

XML Treatment for Zealeuctra wachita

XML Treatment for Zealeuctra warreni
